# Inhibitory Effects of Green Tea and (–)-Epigallocatechin Gallate on Transport by OATP1B1, OATP1B3, OCT1, OCT2, MATE1, MATE2-K and P-Glycoprotein

**DOI:** 10.1371/journal.pone.0139370

**Published:** 2015-10-01

**Authors:** Jana Knop, Shingen Misaka, Katrin Singer, Eva Hoier, Fabian Müller, Hartmut Glaeser, Jörg König, Martin F. Fromm

**Affiliations:** 1 Institute of Experimental and Clinical Pharmacology and Toxicology, Friedrich-Alexander-Universität Erlangen-Nürnberg, Erlangen, Germany; 2 Department of Pharmacology, School of Medicine, Fukushima Medical University, Fukushima, Japan; Fraunhofer Research Institution of Marine Biotechnology, GERMANY

## Abstract

Green tea catechins inhibit the function of organic anion transporting polypeptides (OATPs) that mediate the uptake of a diverse group of drugs and endogenous compounds into cells. The present study was aimed at investigating the effect of green tea and its most abundant catechin epigallocatechin gallate (EGCG) on the transport activity of several drug transporters expressed in enterocytes, hepatocytes and renal proximal tubular cells such as OATPs, organic cation transporters (OCTs), multidrug and toxin extrusion proteins (MATEs), and P-glycoprotein (P-gp). Uptake of the typical substrates metformin for OCTs and MATEs and bromosulphophthalein (BSP) and atorvastatin for OATPs was measured in the absence and presence of a commercially available green tea and EGCG. Transcellular transport of digoxin, a typical substrate of P-gp, was measured over 4 hours in the absence and presence of green tea or EGCG in Caco-2 cell monolayers. OCT1-, OCT2-, MATE1- and MATE2-K-mediated metformin uptake was significantly reduced in the presence of green tea and EGCG (P < 0.05). BSP net uptake by OATP1B1 and OATP1B3 was inhibited by green tea [IC_50_ 2.6% (v/v) and 0.39% (v/v), respectively]. Green tea also inhibited OATP1B1- and OATP1B3-mediated atorvastatin net uptake with IC_50_ values of 1.9% (v/v) and 1.0% (v/v), respectively. Basolateral to apical transport of digoxin was significantly decreased in the presence of green tea and EGCG. These findings indicate that green tea and EGCG inhibit multiple drug transporters in vitro. Further studies are necessary to investigate the effects of green tea on prototoypical substrates of these transporters in humans, in particular on substrates of hepatic uptake transporters (e.g. statins) as well as on P-glycoprotein substrates.

## Introduction

It is well recognized that food ingredients (e.g. furanocoumarins in grapefruit juice) can alter pharmacokinetics and effects of concomitantly administered drugs [1–4]. Green tea (*Camellia sinensis*) and its extracts are commonly used worldwide, in part due to potential positive health effects e.g. regarding prevention of cancer or cardiovascular diseases [5]. The active components of green tea are believed to be the catechins that account for approximately 30% of the tea leaves in dry weight [6]. The major catechin in green tea is epigallocatechin-3-gallate (EGCG) [7].

It appears that green tea and its extract have only limited effects on cytochrome P450-mediated drug metabolism in humans [8, 9]. However, drug disposition and drug effects are also determined by drug transporters [10]. Transporters are involved in transcellular translocation of drugs, e.g. in the intestine, the liver and the kidney. Modification of transporter function by genetic factors (e.g. *SLCO1B1* polymorphisms and simvastatin-induced myopathies) [11] and concomitantly administered drugs [e.g. inhibitors of OCTs/MATEs or inhibitors/inducers of P-glycoprotein (P-gp, *ABCB1*)] contributes to interindividual variability in drug exposure and effects [10]. Organic cation transporter 1 (OCT1, *SLC22A1*) and organic anion transporting polypeptides 1B1 and 1B3 (OATP1B1 and OATP1B3, encoded by *SLCO1B1* and *SLCO1B3*, *respectively*) mediate uptake of drugs from the portal venous blood across the basolateral membrane into hepatocytes. Multidrug and toxin extrusion protein 1 (MATE1, *SLC47A1*) and P-gp are localized in the canalicular membrane of hepatocytes and mediate biliary excretion of their substrates. For the kidney, uptake across the basal membrane of proximal tubular cells by organic cation transporter 2 (OCT2, *SLC22A2*) and efflux via MATE1 and MATE2-K (*SLC47A2*) expressed in the luminal membrane are believed to be important for renal secretion of cationic drugs [10]. Moreover, the efflux pump P-gp limits drug absorption from the intestine due to its expression in the luminal membrane of enterocytes and contributes to renal drug secretion due to its localization in the luminal membrane of proximal tubular cells [10]. Prototypical substrates are bromosulphophthalein and atorvastatin for OATP1B1 and OATP1B3, the frequently used oral antidiabetic drug metformin for OCT1, OCT2, MATE1 and MATE2-K and the cardiac glycoside digoxin for P-gp [10, 12, 13]. Other drug substrates are e.g. pravastatin and rosuvastatin for OATP1B1/OATP1B3, acyclovir, cisplatin, chloroquine and lamivudine for OCTs/MATEs and cyclosporine, dabigatranetexilate, loperamide, quinidine and vincristine for P-glycoprotein [10, 12].

Previous in vitro work suggested that catechins including EGCG might inhibit P-gp [14, 15]. Moreover, Roth et al. showed that uptake of estrone-3-sulfate by the organic anion transporting polypetides OATP1A2 (*SLCO1A2*) and OATP2B1 (*SLCO2B1*) is inhibited by EGCG and (-)-epicatechin-3-gallate (ECG) [16]. We recently showed by in vitro experiments that green tea also inhibits OATP1A2-mediated uptake of the ß-blocker nadolol, possibly explaining in part the pronounced decrease of nadolol plasma concentrations in healthy volunteers after concomitant intake of green tea [17].

The goal of the present in vitro work is to extend our knowledge on green tea and EGCG as inhibitors of drug transporters and to further characterize green tea as potential factor for altered drug disposition in humans. We used cell lines expressing the human transporters OATP1B1, OATP1B3, OCT1, OCT2, MATE1, MATE2-K and P-gp and BSP, atorvastatin, metformin and digoxin as prototypical substrates of the respective transporters.

## Materials and Methods

### Chemicals and materials

[^3^H]Digoxin (20 Ci/mmol) and [^3^H]inulin (18 Ci/mmol) were obtained from American Radiolabeled Chemicals (St. Louis, MO). [^14^C]Metformin (93 Ci/mmol) was obtained from Biotrend (Cologne, Germany). [^3^H]Atorvastatin (20 Ci/mmol) and [^3^H]bromosulphophthalein (BSP, 14 Ci/mmol) were obtained from Hartmann Analytic (Braunschweig, Germany). Unlabeled BSP was purchased from Applichem GmbH (Darmstadt, Germany), unlabeled atorvastatin from Toronto Research Chemicals Inc. (Toronto, Canada). EGCG was kindly provided by Prof. Toshiyuki Kan (Department of Synthetic Organic and Medicinal Chemistry, University of Shizuoka, Japan). Purity of EGCG was higher than 95% (Prof. Asakawa, Department of Synthetic Organic and Medicinal Chemistry, University of Shizuoka, Japan; personal communication). Digoxin, inulin, metformin, and poly-_D_-lysine hydrobromide (PDL) were purchased from Sigma–Aldrich (Taufkirchen, Germany). Zosuquidar was obtained from Selleck Chemicals (Munich, Germany). Minimum essential medium (MEM), and Opti-MEM was purchased from Gibco^®^ (Thermo Fischer Scientific, Waltham, MA). Sodium butyrate was purchased from Merck KGaA (Darmstadt, Germany). As a green tea preparation, Healthya green tea (Kao Corp., Tokyo, Japan) was used that is declared to contain 0.04 kcal/mL calories, 0 g/mL protein, 0 g/mL lipids, 11.1 mg/mL carbohydrates, 0.1 mg/mL sodium, 1.54 mg/mL green tea catechins, and 0.23 mg/mL caffeine. The concentrations of catechins in Healthya green tea were previously determined and were ~80, 240, 130, and 460 μg/ml (i.e. ~1 mM EGCG) for (−)-epicatechin (EC), (−)-epigallocatechin (EGC), (−)-epicatechin-3-gallate (ECG), and EGCG, respectively [17]. All cell culture media supplements were obtained from Life Technologies GmbH (Darmstadt, Germany). Cellstar 12-well cell culture plates and ThinCert^TM^-TC Inserts (12-well, pore size 0.4 μM, translucent) were from Greiner Bio-One GmbH (Frickenhausen, Germany). All other chemicals and reagents, unless stated otherwise, were obtained from Carl Roth GmbH + Co. KG (Karlsruhe, Germany) and were of the highest grade available.

### Cell culture and cloning

Human embryonic kidney 293 (HEK) cells, stably expressing OATP1B1 (HEK-OATP1B1), OATP1B3 (HEK-OATP1B3), OCT1 (HEK-OCT1), OCT2 (HEK-OCT2), and MATE1 (HEK-MATE1) as well as transiently-transfected HEK-MATE2-K cells and the respective vector control (VC) cells were used as previously described (S1 Table) [18–22]. HEK-OATP1B1, HEK-MATE1 and the respective VC cells were cultured in MEM containing 10% heat-inactivated fetal bovine serum (FBS), 800 g/mL geneticin, 100 U/mL penicillin, and 100 μg/mL streptomycin at 37°C and 5% CO_2_, whereas 250 μg/mL hygromycin was used instead of geneticin for HEK-OATP1B3, HEK-OCT1, HEK-OCT2 and the respective VC cells. Parental HEK cells for transient transfection with the MATE2-K plasmid were cultured in the same medium without the addition of geneticin or hygromycin. Caco-2 cells were obtained from American Type Culture Collection (Rockville, MD) and cultured in MEM containing 10% FBS, 1% sodium pyruvate, 100 U/ml penicillin, and 100 mg/ml streptomycin at 37°C and 5% CO_2_. Cells were subcultured by trypsinization using trypsin (0.05%)-EDTA (0.02%) solution. The *SLC47A2*-cDNA encoding human MATE2-K was cloned as previously described using RT-PCR [22]. The cDNA was subcloned into the pcDNA3.1(+) plasmid vector (Life Technologies).

### Uptake studies

Uptake experiments were carried out as previously described [20–23]. Briefly, cells were seeded in 12-well plates coated with poly-D-lysine at a density of 7.0×10^5^ cells/well. After 24 hours, cells were incubated for 24 hours with 10 mM sodium butyrate medium to increase protein expression [24]. Before the uptake experiment, cells were washed with prewarmed uptake buffer (142 mM NaCl, 5 mM KCl, 1 mM K_2_PO_4_, 1.2 mM MgSO_4_, 1.5 mM CaCl_2_, 5 mM glucose, and 12.5 mM HEPES, pH 7.3). Subsequently, cells were incubated with the test solution, containing the substrate atorvastatin (2.5 μM) and [^3^H]BSP for OATP1B1 (0.05 μM) and OATP1B3 (1 μM) or [^3^H]metformin (10 μM) for OCT1 and OCT2 in uptake buffer. HEK cells overexpressing the organic cation/H^+^ antiporter MATE1 and MATE2-K were incubated in a test solution containing uptake buffer adjusted to a pH of 8.0 and [^3^H]metformin (10 μM). Incubation was performed at 37°C for 5 (BSP) or 10 (metformin, atorvastatin) min in the presence or absence of green tea and EGCG. Substrate concentrations and incubation times were chosen based on previous studies in order to obtain high uptake ratios (without inhibitor), which are important in order to generate robust inhibition data. For all cell lines and substrates, transporter functions (i.e. uptake ratios) were in agrement with previous studies. Then cells were washed three times with ice-cold uptake buffer and lysed with 0.2% sodium dodecyl sulfate (SDS). Intracellular accumulation of radioactivity was measured by liquid scintillation counting (Tricarb2 800, Perkin Elmer Life Sciences GmbH, Rodgau-Jügesheim, Germany). Protein concentration of each sample was determined by bicinchoninic acid assay (Pierce BCA protein assay kit, Thermo Scientific, Rockford, IL, USA).

### Uptake experiments using HEK cells transiently overexpressing MATE2-K

Experiments were performed as previously described [22]. In brief, parental HEK cells were seeded on poly-D-lysine-coated (0.1 mg/ml) 12-well plates with a seeding density of 5 x 10^5^ cells or 3.5 x 10^5^ cells for transfection with MATE2-K or empty control vector, respectively. Twenty-four hours after seeding, cells were transfected with 1.5 μg/well of the corresponding plasmid using Lipofectamine 2000 and Optimem I according to the manufacturer´s protocol. Forty-eight hours after seeding, the medium was exchanged with cell culture medium. Seventy-two hours after seeding, uptake experiments were performed as described earlier.

### Transcellular transport studies in Caco-2 cell monolayers

Cells were plated on Transwell filters. Transport experiments were performed on day 7 after plating. About 1 hour prior to the start of the transport experiment, the medium in each compartment was replaced by Opti-MEM. For transport experiments, the Opti-MEM in each compartment was then replaced with 800 μL serum-free medium with addition of a radiolabeled substrate on the basal or the apical side of the monolayer. During the incubation at 37°C, the amount of substrate appearing in the opposite compartment (basal or apical) after 1, 2, 3, and 4 hours was measured in aliquots. Donor solutions were prepared for permeability comparators of a positive control substrate [^3^H]digoxin (5 μM) and a low permeability control [^3^H]inulin (50 μM). The bidirectional permeability values were also determined in the presence of zosuquidar (1 μM), green tea [1% (v/v) and 10% (v/v)] and EGCG (1 μM) present in both donor and receiver chambers. Experiments were conducted only with wells that had a transepithelial resistance of >200 Ω measured by Millicell^®^ ERS-2 Voltohmmeter (Merck Millipore, Darmstadt, Germany) after correction for the resistance obtained in control blank wells. The radioactivity was measured by liquid scintillation counting (Tricarb2 800, Perkin Elmer Life Sciences GmbH, Rodgau-Jügesheim, Germany). Transcellular, basal to apical translocation of inulin was routinely checked and was in average below 1.6% / hour.

### Data analysis and statistics

For each concentration and time point four experiments were performed. Substrate uptake was normalized with respect to protein concentration of the cell lysate. Net transport was obtained by subtraction of the transport by VC cells from that by transporter-overexpressing cells. For calculation of half-maximal inhibitory concentrations (IC_50_), the net uptake was converted to percent of uptake in the absence of inhibitor. These values were averaged, and the mean values were fitted to nonlinear regression using GraphPad Prism (version 5.01 for Windows, 2007; GraphPad Software Inc., San Diego, USA). For Caco-2 assays, apical-to-basal and basal-to-apical transcellular transport was calculated as percentage of initial concentration of compound applied to the donor chamber. Data were analyzed by twotailed unpaired Student’s t-test or by one-way ANOVA with Bonferroni test for selected comparisons using GraphPad Prism (GraphPad software, Inc.) as appropriate. Differences were regarded as statistically significant with *P* < 0.05. Data are presented as the mean ± standard error of the mean (SEM).

## Results

### Effect of green tea on cation transporters

**Fig 1:**
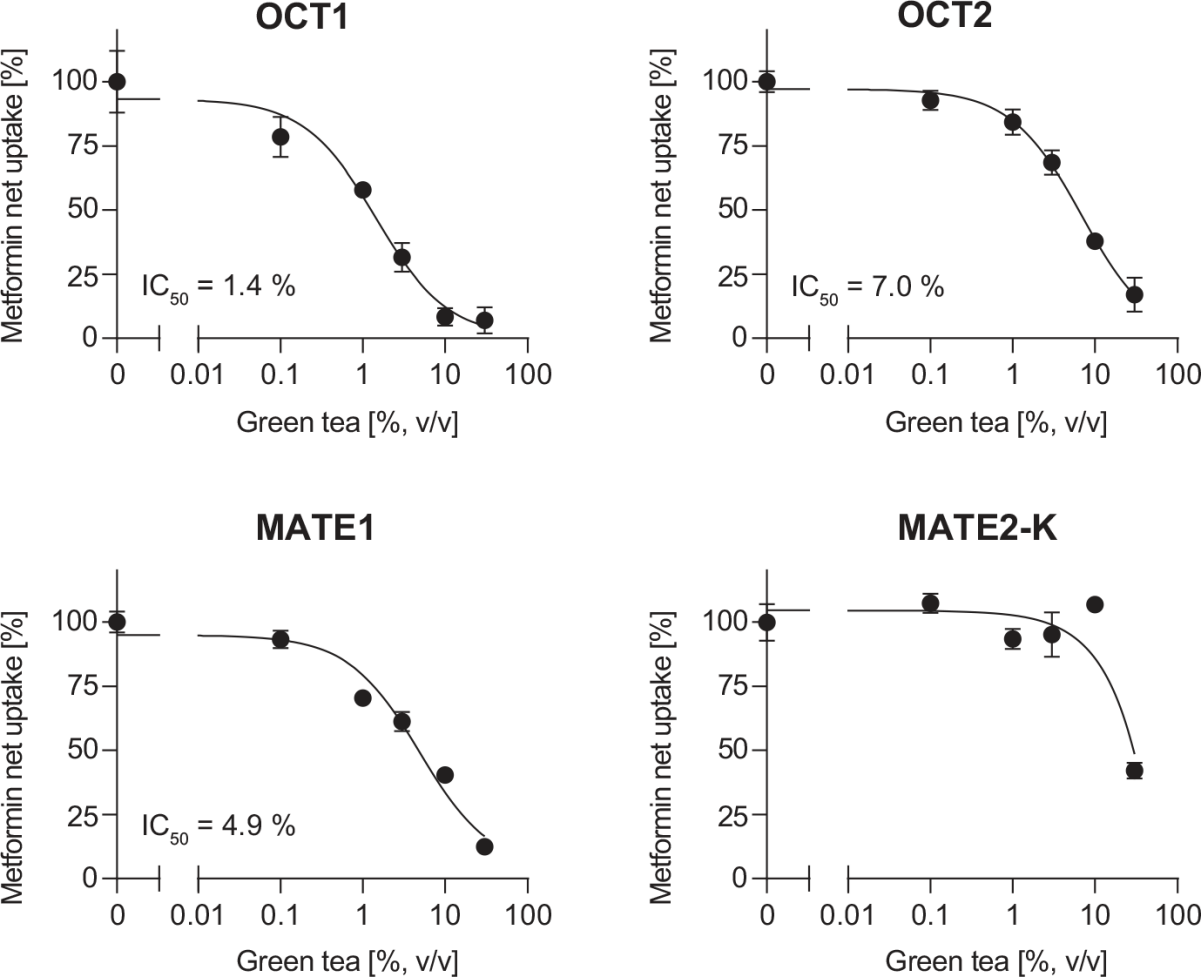
Effect of green tea on OCT1-, OCT2-, MATE1- and MATE2-K-mediated metformin net transport. Data are shown as mean net uptake ± S.E.M.

The uptake of metformin was measured in the absence or presence of green tea [0.1 to 30% (v/v)]. Metformin uptake was inhibited in a concentration dependent manner in the presence of green tea with IC_50_ values of 1.4% (v/v) (95% CI: 0.70–3.0%) and 7.0% (v/v) (95% CI: 3.8–12%) for OCT1 and OCT2, respectively (Fig 1 and S1 Fig). The inhibitory potency of green tea on metformin uptake was stronger for MATE1 compared to MATE2-K. While IC_50_ of green tea was 4.9% (v/v) (95% CI: 2.9–8.3%) for inhibition of MATE1-mediated metformin transport, the IC_50_ value for MATE2-K-mediated metformin transport could not be calculated (Fig 1 and S1 Fig).

### Effect of EGCG on cation transporters

**Fig 2:**
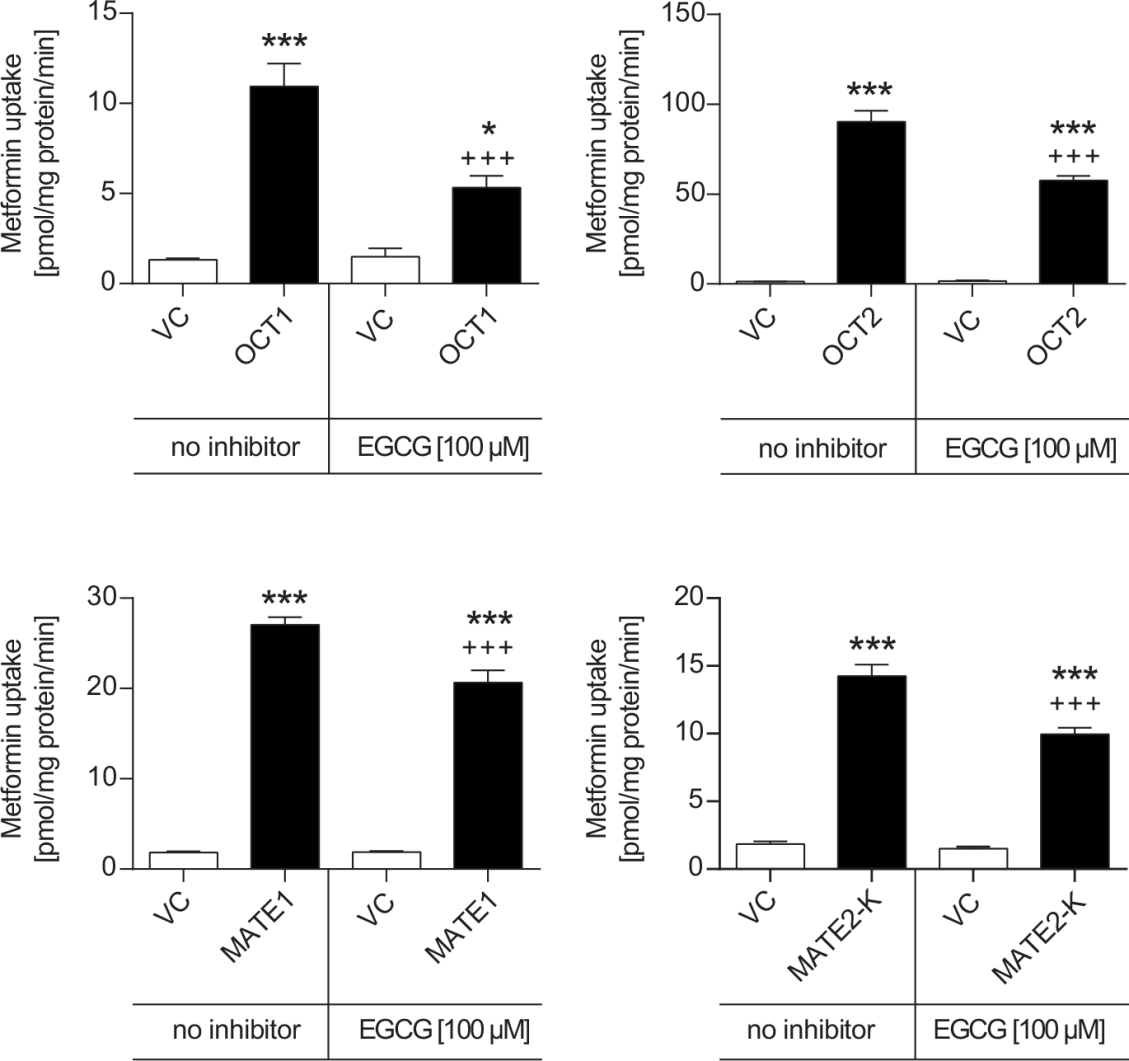
Effect of EGCG on OCT1-, OCT2-, MATE1- and MATE2-K-mediated metformin transport. Data are shown as mean ± S.E.M. VC vector control. *** p< 0.001, * p<0.05 vs. VC, +++ p<0.001 vs. corresponding transport without inhibitor.

Transporter-mediated uptake of metformin was measured in the absence or presence of 100 μM EGCG (Fig 2). OCT1-mediated metformin uptake was 8.3-fold higher (p<0.001) compared to VC cells in the absence of EGCG. In the presence of EGCG the OCT1-mediated metformin uptake was only 3.6-fold higher (p<0.05) compared to VC cells. OCT1-mediated metformin net uptake (i.e. uptake into transporter-transfected cells minus uptake into vector control cells) was significantly reduced by EGCG to 40% of metformin net uptake without EGCG (p<0.01).

OCT2-mediated metformin uptake without EGCG was 69-fold higher (p<0.001), while in the presence of EGCG the uptake was only 39-fold higher compared to VC cells (p<0.001). OCT2-mediated metformin net uptake was significantly reduced by EGCG to 63% of metformin net uptake without EGCG (p<0.01).

The MATE1- and MATE2-K-mediated metformin uptake was 14- and 7.6-fold higher compared to VC cells, respectively (p<0.001). In the presence of EGCG the metformin uptake was decreased to 11-fold for MATE1- and 6.5-fold for MATE2-K-mediated transport (p<0.001). For both, MATE1 and MATE2-K the net transport in the presence of EGCG was reduced to 74% and 68%, respectively, compared to the uptake in absence of EGCG (p<0.01 and p<0.05, respectively).

### Effect of green tea on OATP1B1- and OATP1B3- mediated transport of BSP and atorvastatin

**Fig 3:**
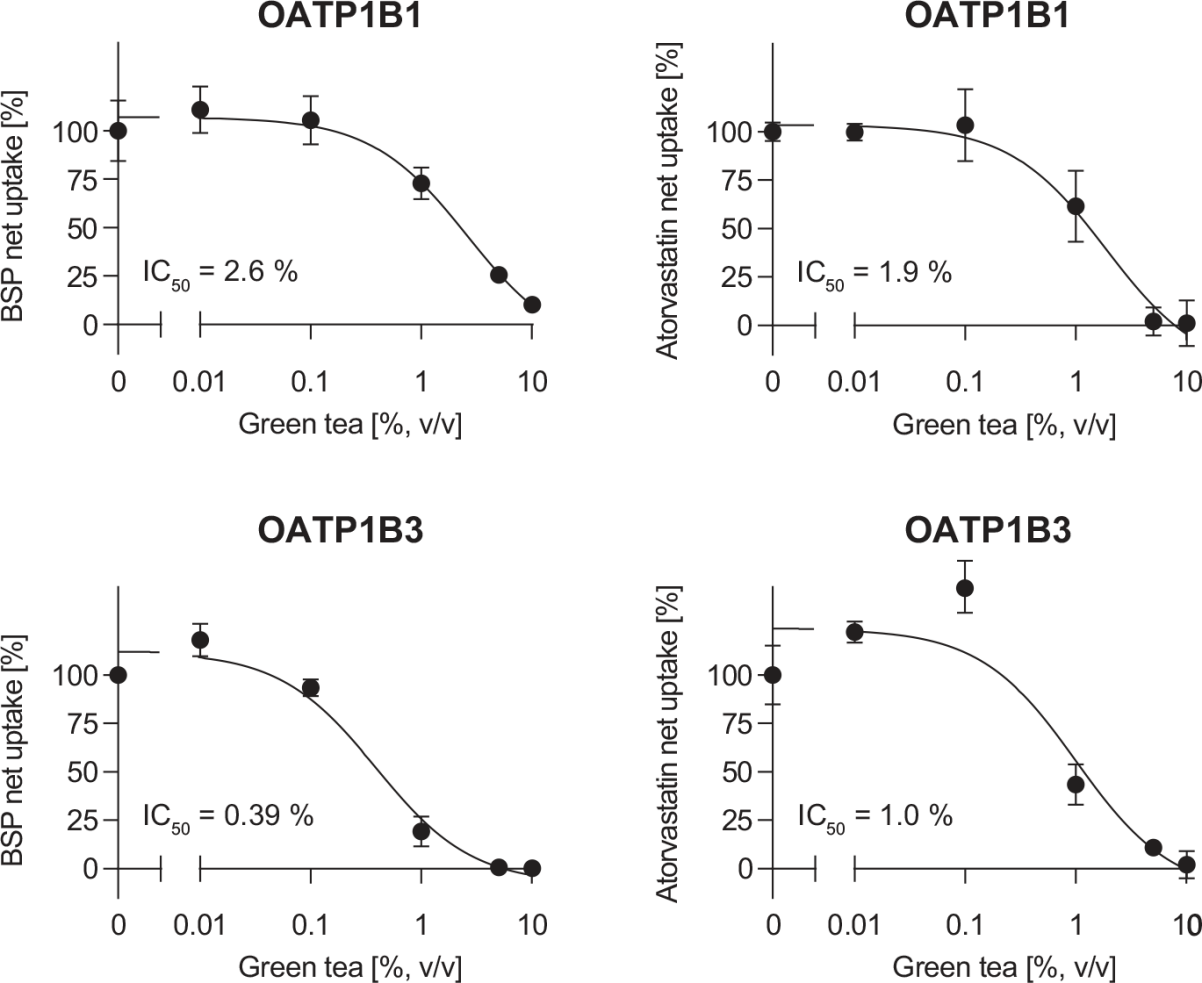
Effect of green tea on OATP1B1- and OATP1B3-mediated BSP and atorvastatin net transport. Data are shown as mean net uptake ± S.E.M. VC vector control.

Cells were incubated with the substrates BSP or atorvastatin in the absence or presence of green tea (0.01 to 10%). The addition of green tea caused a concentration-dependent inhibition of BSP and atorvastatin net uptake in HEK-OATP1B1 and HEK-OATP1B3 cells (Fig 3 and S2 Fig). IC_50_ values of green tea for BSP uptake were 2.6% (v/v) (95% CI: 0.75–9.2%) and 0.39% (v/v) (95% CI: 0.21–0.71%) for OATP1B1 and OATP1B3 cells, respectively (Fig 3 and S2 Fig). For inhibition of atorvastatin uptake in the presence of green tea IC_50_ values of 1.9% (v/v) (95% CI: 0.52–6.7%) and 1.0% (v/v) (95% CI: 0.32–3.2%) were calculated for OATP1B1 and OATP1B3 cells, respectively.

### Effect of EGCG on OATP1B1- and OATP1B3-mediated transport

**Fig 4:**
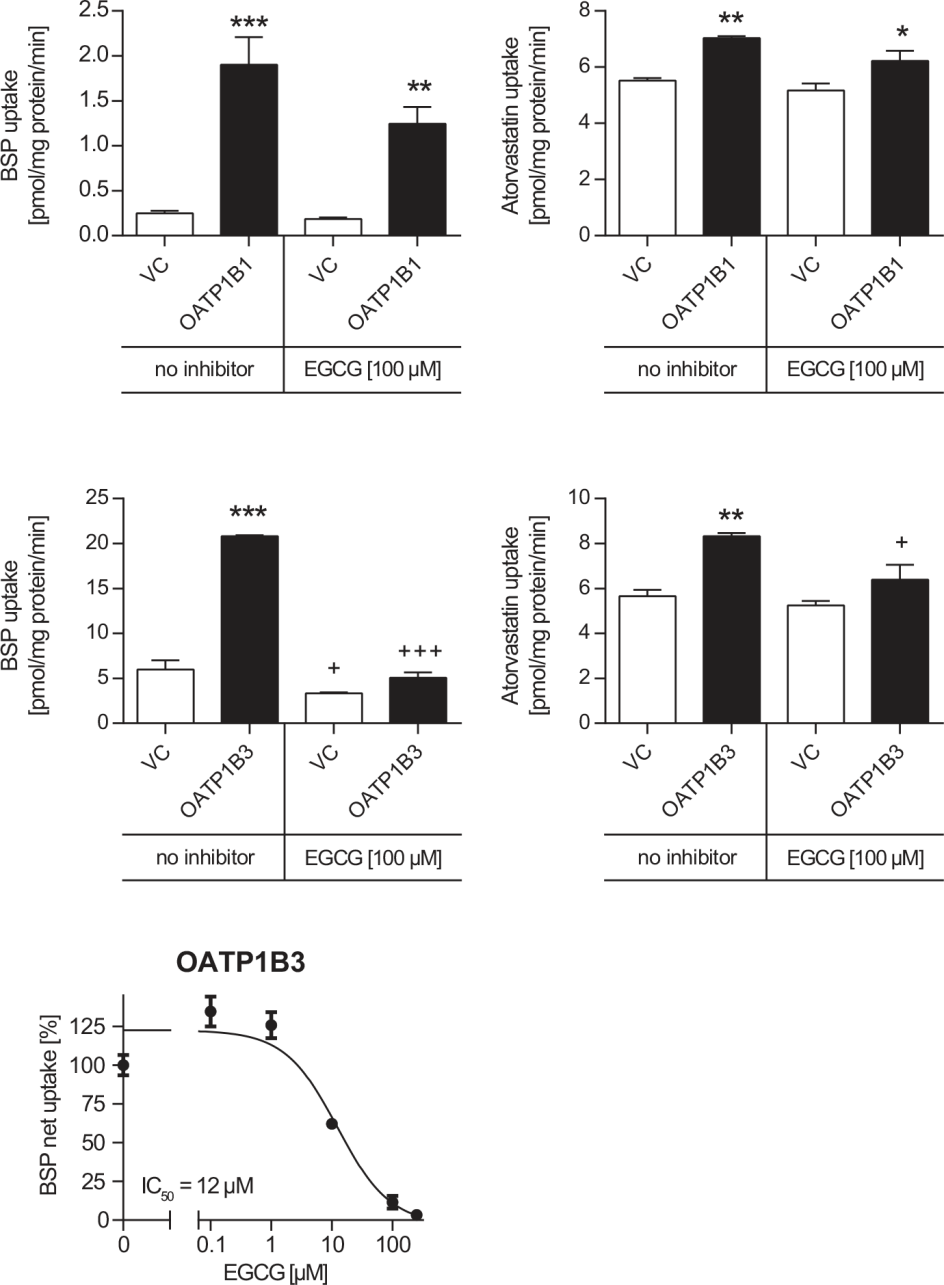
Effect of EGCG on OATP1B1- and OATP1B3-mediated BSP and atorvastatin transport. Data are shown as mean ± S.E.M. *** p< 0.001, ** p<0.01, * p<0.05 vs. VC, + p<0.05, +++ p<0.001 vs. corresponding transport without inhibitor.

The uptake of BSP or atorvastatin was measured in the absence or presence of EGCG [100 μM (Fig 4)]. OATP1B1-mediated uptake of BSP or atorvastatin was 7.7-fold (p<0.001) and 1.3-fold (p<0.01) higher compared to VC cells, respectively. In the presence of 100 μM EGCG the OATP1B1-mediated BSP or atorvastatin uptake was 6.7-fold (p<0.01) and 1.2-fold (p<0.05) higher compared to VC cells. OATP1B1-mediated BSP and atorvastatin net uptake (i.e. uptake into transporter-transfected cells minus uptake into vector control cells) were reduced by EGCG to 64% (n.s.) and 69% (p<0.05), respectively, of net uptake without EGCG.

OATP1B3-mediated BSP or atorvastatin uptake was 3.5-fold (p<0.001) and 1.5-fold higher (p<0.01) compared to VC cells, respectively. In the presence of EGCG the uptake was only 1.5-fold and 1.2-fold higher compared to VC cells. OATP1B3-mediated BSP and atorvastatin net uptake were reduced by EGCG to 12% (p<0.001) and 43% (n.s.), respectively, of net uptake without EGCG. The IC_50_ value for inhibition of OATP1B3-mediated BSP uptake by EGCG was 12 μM (Fig 4).

### Effect of green tea and EGCG on P-gp-mediated transport of digoxin

**Fig 5:**
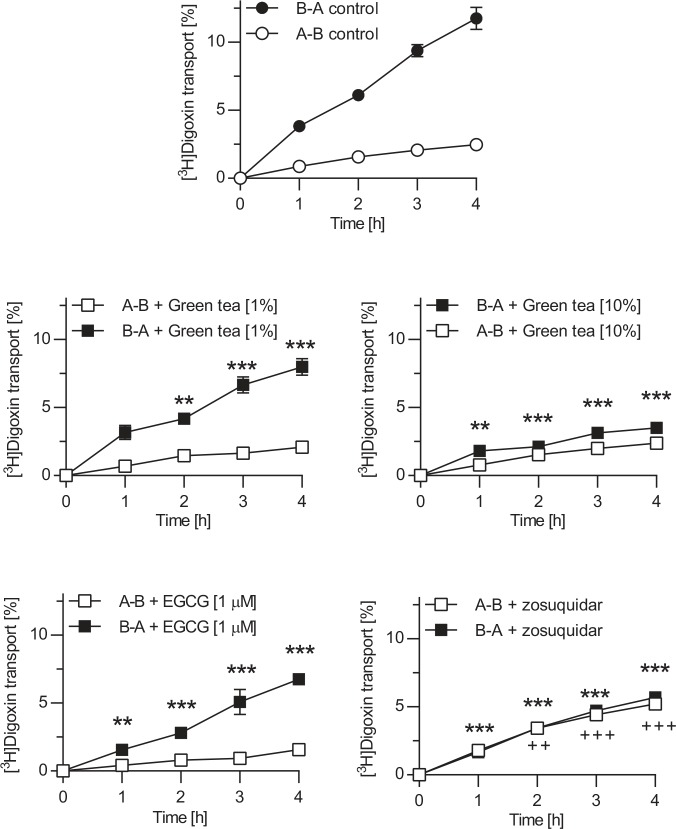
Effect of green tea and EGCG on transepithelial transport of digoxin across Caco-2 monolayers. Digoxin (5 μM) was applied to the basal or apical compartment, and percentage of digoxin transported in the opposite compartment at defined time points was measured. Experiments were conducted in the absence of an inhibitor (top) or in the presence of green tea (1% (v/v) and 10%), EGCG (1 μM) or the P-glycoprotein inhibitor zosuquidar (1 μM). Data are expressed as mean ± S.E.M. A-B apical to basal translocation, B-A basal to apical translocation; **/^++^ p<0.01, ***/^+++^ p<0.001 vs. corresponding control without inhibitor.

To investigate the effect of green tea and its main catechin EGCG on P-gp-mediated transport, we performed transcellular transport studies in monolayers of Caco-2 cells using digoxin as P-gp substrate. The transport of digoxin in the Caco-2 cell system was characterized in the absence and presence of EGCG, green tea and the P-gp inhibitor zosuquidar (Fig 5). In the absence of inhibitor, the polarized digoxin transport was markedly higher in the basal-to-apical transport direction (3.2% transported digoxin/h) than in the opposite direction (0.74%/h). The presence of green tea significantly decreased the basal-to-apical digoxin transport to 2.4%/h for 1% (v/v) green tea. 10% (v/v) green tea abolished polarized digoxin transport. EGCG (1μM) also significantly inhibited the basal-to-apical digoxin transport to 1.6%/h. As expected, the addition of the known P-gp inhibitor zosuquidar abolished the polarized transcellular transport of digoxin.

## Discussion

We previously reported that repeated consumption of green tea markedly decreased the plasma concentration and effect on blood pressure of the OATP1A2 substrate nadolol [17]. To further analyze the inhibitory potency of green tea on drug transport we analyzed in the present study the effect of green tea and its main catechin EGCG on seven important drug transporters expressed in intestine, liver and kidney. Our results demonstrate that green tea inhibits in vitro transport of prototypical substrates of all seven drug transporters investigated (OCT1, OCT2, MATE1, MATE2-K, OATP1B1, OATP1B3, P-gp). Additionally we found inhibitory effects of the major green tea catechin EGCG on all transporters tested with the highest in vitro inhibitory potency for OATP1B3.

The inhibitory effect of green tea on the cationic drug transporters was most pronounced for OCT1 with an IC_50_ value for inhibition of metformin transport of 1.4% (v/v) followed by MATE1 and OCT2 with IC_50_ values of 4.9% (v/v) and 7.0% (v/v), respectively. The major catechin in green tea EGCG also significantly inhibited cationic drug transporters. In line with the green tea inhibition, EGCG had also the highest inhibitory potency at the OCT1-mediated metformin transport compared to OCT2, MATE1 and MATE-2K. Furthermore, in vitro inhibition of the hepatic uptake transporters OATP1B1 and OATP1B3 by green tea was also shown. The IC_50_ values for inhibition of OATP1B1- and OATP1B3-mediated BSP or atorvastatin transport were in a range between 0.39% (v/v) and 2.6% (v/v). In line with the data on OATP1B1- and OATP1B3-inhibition by green tea, EGCG inhibited more potently OATP1B3-mediated transport compared to OATP1B1-mediated transport.

The concentration of EGCG in the green tea preparation used in our experiments is 0.46 mg/ml [17]. Therefore, a 1:10 dilution of green tea (10%) contains 100 μM EGCG. A comparison of the transport activity in the presence of either 10% (v/v) green tea or 100 μM EGCG shows a higher inhibitory efficacy for green tea than for 100 μM EGCG. For example in the presence of 10% (v/v) green tea the MATE1-mediated metformin transport was reduced to 40%, while in the presence of 100 μM EGCG the metformin uptake was about 74% compared to the absence of inhibitor. The likely explanation for this observation is the presence of further catechins in green tea with inhibitory effects on drug transporters. Accordingly, Roth et al. previously showed an inhibitory effect of epicatechin gallate (ECG) on OATP1A2-, OATP1B1-, and OATP2B1-mediated estrone-3-sulfate transport [16].

The major question is, what do these in vitro data mean for potential effects of green tea on disposition of drugs in humans? OCT2 is expressed in the basal membrane of renal proximal tubular cells. Thus, sufficiently high unbound inhibitor concentrations need to be present in the systemic circulation in order to inhibit OCT2. MATE1 and MATE-2K are localized in the luminal membrane of renal proximal tubular cells. Since intracellular concentrations of potential inhibitors in humans are unknown, systemic plasma concentrations are used as indicators of potential inhibitor effects on MATEs. EGCG plasma concentrations in humans reach low μM concentrations [9, 17]. Since 100 μM EGCG inhibited OCT2-, MATE1-, and MATE-2K-mediated metformin transport less than 50%, we consider it unlikely that green tea has a clinically meaningful effect on transporter-mediated, renal secretion of cationic drugs. To the best of our knowledge, plasma protein binding of EGCG in humans has not been determined. However, EGCG plasma protein binding in rats is higher than 90% [25, 26] further arguing against a relevant in vivo inhibition of renal cation transporters by EGCG.

The hepatic uptake transporters OCT1, OATP1B1 and OATP1B3 were inhibited by green tea with IC_50_ values of 0.39 to 2.6%. The IC_50_ value for inhibition of OATP1B3-mediated BSP uptake by EGCG was 12 μM and 100 μM EGCG inhibited OATP1B3-mediated atorvastatin transport by more than 50%. Based on our clinical study [17] we estimate, using the formula of Ito et al. [27], that total EGCG concentrations in portal venous blood can reach approximately 50 μM. Assuming a 90% plasma protein binding in humans, free EGCG concentrations could be approximately 5 μM in human portal venous blood. Since green tea contains also other ingredients which inhibit drug transporters [16], it cannot be excluded based on the currently available data that green tea affects transporter-mediated uptake into hepatocytes. Interestingly, green tea did not affect disposition of the OATP substrate simvastatin in Italian healthy volunteers, but caused a significant increase in simvastatin acid plasma concentrations in Japanese volunteers [28].

In our opinion, inhibition of intestinal P-gp by green tea could be the clinically most relevant mechanism underlying an increased exposure of P-gp substrates after green tea consumption. In our studies, a 1:100 dilution of green tea already had significant effects on digoxin basal to apical translocation. A 1:10 dilution of green tea completely abolished polarized digoxin transport. Moreover, 1 μM EGCG significantly inhibited digoxin basal to apical translocation, while commercial green tea can have EGCG concentrations up to 1 mM [17, 29]. Several studies previously investigated the impact of green tea or green tea catechins on P-gp function using different cellular models and P-gp substrates. For example, Fleisher et al. reported no influence of four green tea catechins including EGCG on P-gp-mediated dasatinib efflux in LLC-PK1/Pgp cells at a concentration of 50 μM [29]. On the other hand, Jodoin et al. showed that EGCG (100 μM) as well as the P-gp inhibitor PSC-833 (10 μM) more than doubled apical-to-basal translocation of the P-gp substrate vinblastine after administration of vinblastine to the apical compartment using Caco-2 cells [15]. In line with these findings, Engdal and Nilsen [30] reported a significant inhibition of P-gp-mediated digoxin transport by green tea in Caco-2 cells. To the best of our knowledge, no clinical studies on the influence of green tea on pharmacokinetics of recommended “probe drugs” for P-gp function such as digoxin or dabigatran have been performed.

It is interesting to note that EGCG inhibited in our study a relatively broad spectrum of partially structurally quite distinct transporters. There are several examples of other compounds which inhibits multiple, structurally distinct transporters. One of these compounds is cyclosporine, which inhibits multiple transporters. Since the molecular mechanisms of substrate transport by these transporters as well as inhibitor interaction with these transporters are incompletely understood, there is currently no established theory, why certain compounds such as EGCG inhibit multiple transporters, whereas other compounds are relatively specific.

Further in vitro studies are required to test the interaction of catechins other than EGCG on transporter function. Moreover, green tea, EGCG and other catechins in green tea might also alter the function of other transporters not studied in this work (e.g. BCRP, MRP2). Further studies should also include primary cells expressing multiple transporters, e.g. human hepatocytes, in order to obtain additional information regarding the effect of green tea and green tea catechins on transport in cellular systems endogenously expressing multiple transporters.

In conclusion we have demonstrated the in vitro inhibitory effect of green tea and EGCG on OCT1, OCT2, MATE1, MATE2-K, OATP1B1, OATP1B3 and P-gp. The inhibitory effect of green tea exceeds the effect of its main catechin EGCG alone. Additional inhibitory effects of other catechins could be one explanation. Based on our findings, we consider an effect of green tea on the function of renal cation transporters in humans as unlikely. Studies in humans should be performed to investigate the effects of green tea on pharmacokinetics of prototypical substrates of hepatic uptake transporters. Finally, based on our data, inhibition of intestinal P-gp by green tea could affect disposition of P-gp substrates in humans.

## Supporting Information

S1 Fig Effect of green tea on OCT1-, OCT2-, MATE1- and MATE2-K-mediated metformin transport. Data are shown as mean ± S.E.M. Open circle, vector control cells; closed circle, transporter-transfected cells.
(EPS)

S2 Fig Effect of green tea on OATP1B1- and OATP1B3-mediated BSP and atorvastatin transport. Data are shown as mean ± S.E.M. Open circle, vector control cells; closed circle, transporter-transfected cells.
(EPS)

S1 Table Transporter-transfected HEK cell lines used in this study. (DOCX)

## Abbreviations

BSP: bromosulphophthalein
EGCG: (–)-epigallocatechin gallate
HEK: human embryonic kidney
MATE: multidrug and toxin extrusion protein
OATP: organic anion transporting polypeptide
OCT: organic cation transporter
P-gp: P-glycoprotein
