# Supplementary material for: Inhibitory Effects of Green Tea and (–)-Epigallocatechin Gallate on Transport by OATP1B1, OATP1B3, OCT1, OCT2, MATE1, MATE2-K and P-Glycoprotein
Source: PLoS One. 2015 Oct 1;10(10):e0139370. doi: 10.1371/journal.pone.0139370 (PMC4591125; doi:10.1371/journal.pone.0139370)
Supplement: S1 Table — (DOCX) [file pone.0139370.s003.docx]

**Table S1 Transporter-transfected HEK cell lines used in this study**

| Transporter | Plasmid | cDNA | Antibiotic | Reference |
| --- | --- | --- | --- | --- |
| OCT1 | pcDNA3.1/Hygro(-) | own synthesis | hygromycin | [[1](#_ENREF_1)] |
| OCT2 | pcDNA3.1/Hygro(-) | own synthesis | hygromycin | [[2](#_ENREF_2)] |
| MATE1 | pcDNA3.1(+) | own synthesis | geneticin | [[3](#_ENREF_3), [4](#_ENREF_4)] |
| MATE2-K | pcDNA3.1(+) | own synthesis | - (transient transfections) | [[4](#_ENREF_4)] |
| OATP1B1 | pcDNA3.1(+) | Prof. D. Keppler (DKFZ Heidelberg, Germany) | geneticin | [[5](#_ENREF_5)] |
| OATP1B3 | pcDNA3.1/Hygro(-) | Prof. D. Keppler (DKFZ Heidelberg, Germany) | hygromycin | [[5](#_ENREF_5)] |

1. Huber S, Huettner JP, Hacker K, Bernhardt G, König J, Buschauer A. Esters of bendamustine are by far more potent cytotoxic agents than the parent compound against human sarcoma and carcinoma cells. PloS one. 2015; *in press*.

2. Zolk O, Solbach TF, König J, Fromm MF. Structural determinants of inhibitor interaction with the human organic cation transporter OCT2 (SLC22A2). Naunyn Schmiedebergs Arch Pharmacol. 2009;379(4):337-48. Epub 2008/11/13. doi: 10.1007/s00210-008-0369-5. PMID: 19002438.

3. König J, Zolk O, Singer K, Hoffmann C, Fromm MF. Double-transfected MDCK cells expressing human OCT1/MATE1 or OCT2/MATE1: determinants of uptake and transcellular translocation of organic cations. Br J Pharmacol. 2011;163(3):546-55. Epub 2010/10/05. doi: 10.1111/j.1476-5381.2010.01052.x. PMID: 20883471; PubMed Central PMCID: PMC3101617.

4. Müller F, König J, Hoier E, Mandery K, Fromm MF. Role of organic cation transporter OCT2 and multidrug and toxin extrusion proteins MATE1 and MATE2-K for transport and drug interactions of the antiviral lamivudine. Biochem Pharmacol. 2013;86(6):808-15. Epub 2013/07/24. doi: 10.1016/j.bcp.2013.07.008. PMID: 23876341.

5. Seithel A, Eberl S, Singer K, Auge D, Heinkele G, Wolf NB, et al. The influence of macrolide antibiotics on the uptake of organic anions and drugs mediated by OATP1B1 and OATP1B3. Drug Metab Dispos. 2007;35(5):779-86. Epub 2007/02/14. doi: 10.1124/dmd.106.014407. PMID: 17296622.
